# Comparative genomics reveals molecular features unique to the songbird lineage

**DOI:** 10.1186/1471-2164-15-1082

**Published:** 2014-12-13

**Authors:** Morgan Wirthlin, Peter V Lovell, Erich D Jarvis, Claudio V Mello

**Affiliations:** Department of Behavioral Neuroscience, Oregon Health & Science University, Portland, OR 97214 USA; Department of Neurobiology, Howard Hughes Medical Institute and Duke University Medical Center, Durham, NC 27710 USA

**Keywords:** Songbird, Novel gene family expansion, Evolution, Vocal learning, Zebra finch

## Abstract

**Background:**

Songbirds (oscine Passeriformes) are among the most diverse and successful vertebrate groups, comprising almost half of all known bird species. Identifying the genomic innovations that might be associated with this success, as well as with characteristic songbird traits such as vocal learning and the brain circuits that underlie this behavior, has proven difficult, in part due to the small number of avian genomes available until recently. Here we performed a comparative analysis of 48 avian genomes to identify genomic features that are unique to songbirds, as well as an initial assessment of function by investigating their tissue distribution and predicted protein domain structure.

**Results:**

Using BLAT alignments and gene synteny analysis, we curated a large set of Ensembl gene models that were annotated as novel or duplicated in the most commonly studied songbird, the Zebra finch (*Taeniopygia guttata*), and then extended this analysis to 47 additional avian and 4 non-avian genomes. We identified 10 novel genes uniquely present in songbird genomes. A refined map of chromosomal synteny disruptions in the Zebra finch genome revealed that the majority of these novel genes localized to regions of genomic instability associated with apparent chromosomal breakpoints. Analyses of *in situ* hybridization and RNA-seq data revealed that a subset of songbird-unique genes is expressed in the brain and/or other tissues, and that 2 of these (*YTHDC2L1* and *TMRA*) are highly differentially expressed in vocal learning-associated nuclei relative to the rest of the brain.

**Conclusions:**

Our study reveals novel genes unique to songbirds, including some that may subserve their unique vocal control system, substantially improves the quality of Zebra finch genome annotations, and contributes to a better understanding of how genomic features may have evolved in conjunction with the emergence of the songbird lineage.

**Electronic supplementary material:**

The online version of this article (doi:10.1186/1471-2164-15-1082) contains supplementary material, which is available to authorized users.

## Background

**Figure 1:**
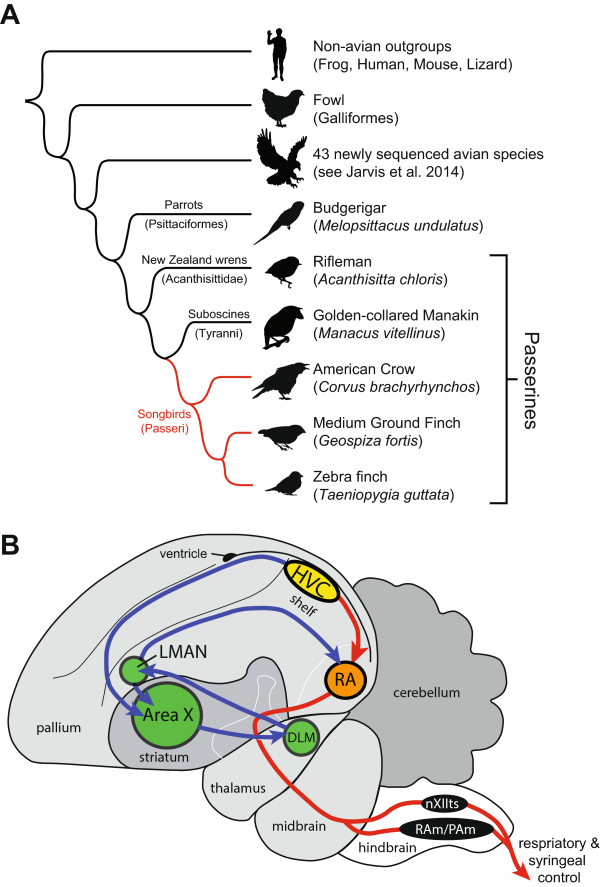
**Phylogenetic analysis used to identify the set of novel genes unique to songbirds (oscine passerines). (A)** Schematic diagram summarizing phylogenetic relationships among organisms included in genomic analyses, not to scale. Full details on genomes included in these analyses are reported in main avian phylogenomics and comparative genomics papers [34, 35]. **(B)** Simplified schematic of the Zebra finch nuclei specialized for vocal learning and their connections. Red projection: the posterior vocal motor pathway for vocal control, originating in HVC, in yellow, continuing to RA, in orange, to the hindbrain vocal motor nuclei, in black. Blue projection: the anterior forebrain pathway for vocal learning, originating in HVC, which projects to a set of interconnected nuclei (Area X, LMAN, DLM) analogous to mammalian cortical-basal ganglia-thalamo-cortical loops for somatosensory learning. Abbreviations: DLM, medial part of the dorsal lateral nucleus of the thalamus; LMAN, lateral magnocellular nucleus of the anterior nidopallium; HVC, proper name; nXIIts, tracheosyringeal portion of the hypoglossal nucleus; PAm/RAm, nucleus para-ambiguus/retroambiguus; RA, robust nucleus of the arcopallium.

Passeriformes are the largest tetrapod order, with over 5,700 species found across the globe [1]. The vast majority of these are oscine passerines, or songbirds (suborder: Passeri), with far fewer species of suboscines (suborder: Tyranni) and just 2 extant species of basal New Zealand wrens (suborder: Acanthisitti) (Figure 1A). Passerines are distinguished by a number of traits including their distinctive foot anatomy adapted for perching, an altricial pattern of offspring growth, and an exceptionally high metabolic rate [2, 3]. Unique to songbirds is the complex syringeal morphology and underlying brain circuitry for vocal learning, the ability to imitate a tutor’s song transmitted across successive generations [4, 5]. Vocal learning provides the basis for human speech acquisition, and is exceedingly rare in the animal kingdom [6, 7]. Aside from humans, it has been convincingly found in few other mammalian groups (bats [8, 9], cetaceans [10, 11], and possibly elephants [12] and pinnipeds [13, 14]), where the underlying brain circuitry and mechanisms are unknown, and just 3 of more than 30 extant orders of birds (songbirds, parrots, and hummingbirds [6, 15]). Notably, interconnected forebrain circuitry involved in the perception and production of learned vocalizations has been discovered in all three avian vocal learning groups [16–24]. Best studied in songbirds, this circuitry includes cortical-like, basal ganglia, and thalamic nuclei (Figure 1B, for reviews see [6, 25, 26]).

The Zebra finch (Passeriformes: *Taeniopygia guttata*), a songbird native to Australia, has been an important model for understanding the neurobiology of sensorimotor integration, memory, and sexual differentiation, among other functions, as well as being the primary model for studying the neural basis of vocal learning. The completion of the Zebra finch genome [27] made it possible to search for genomic features that might be unique to songbirds. The emergence of novel genes has been shown to provide a genetic substrate for lineage-specific adaptations and the evolution of new functional traits [28, 29]. It is thus possible that novel genes might also be associated with the evolution of characteristic songbird traits like vocal learning. The initial comparative analysis between Zebra finch and chicken (Galliformes: *Gallus gallus*), a previously sequenced, non-learning species [30], led to the identification of candidate novel gene duplications and expansions in Zebra finches [27]. However, without additional genomes that span the large phylogenetic distance between Galliformes and Passeriformes, it was impossible to conclude whether these features are specific to Zebra finch, or originated more basally in finches, oscines, Passeriformes, or elsewhere within Neoaves. Moreover, it has not been determined whether these genomic features are associated with vocal learning and related circuitry, or with other phenotypic differences between oscines and non-oscines. In the few cases where this question has been examined, there is only very limited evidence of regional or differential gene expression in the brain [27, 31].

A requirement for accurate comparative genomics are well-curated sets of 1-to-1 orthologs among the organisms being compared. Obtaining the true set of orthologs is an ongoing problem, and many genomes remain partial and/or insufficiently annotated, in part due to errors by *ab initio* gene predictive algorithms to identify all exons of a given ortholog, even when these are present in the genome assembly [32]. These issues have led to erroneous annotations of Zebra finch models as novel, non-detection of orthologs that are in fact present in the assembly, and erroneous conclusions about gene duplications and expansions [33]. Thus, the search for genomic features unique to Zebra finches, or more broadly to songbirds, and that may relate to their distinct traits, is still very incomplete.

Here, we have curated the Zebra finch Ensembl gene models annotated as novel or duplicated, as well as several previously identified novel expansions in the Zebra finch genome (Supplementary Figure Three in [27]), and then determined their presence or absence in 45 recently sequenced, high-coverage (30–120X) genomes spanning the avian phylogeny (phylogeny and genomes described in [34, 35]). As a result, we have identified a set of genes that are clearly novel in songbirds, and provide evidence for chromosomal rearrangement as a potential mechanism for their origin. We also show that some of these genes are expressed in the brain, and that a small subset are differentially expressed in song nuclei relative to surrounding areas, representing molecular specializations of the songbird vocal control system; others are expressed in various non-neural tissues. In contrast, we found that several hundred putative previously reported novel songbird gene models represent artefacts, previously known genes, or genes that are present in non-songbird species. Our results provide an approach for improved genome annotation, as well as identifying novel targets for investigating genes unique to a lineage or trait, including vocal learning and its associated brain circuitry in songbirds.

## Results

We implemented a comprehensive and exhaustive annotation pipeline to identify genes that evolved in songbirds subsequent to their divergence from all other birds. Specifically, we first focused on the best-annotated songbird species, the Zebra finch, retrieving all Zebra finch Ensembl gene models annotated as novel, duplicated, or expanded (>7,000 models, e59), and removing models Ensembl considered to have orthologs in other species (n = 5,459), as well as those mapped to chromosome Unknown (n = 1,179), which were likely to represent allelic variants. The mRNA and protein sequences of the remaining models (n = 876) were BLAT-aligned to chicken, currently the best-annotated avian species, and Zebra finch, and synteny for all hits of sufficiently high score was verified to identify conserved orthologs. Loci present in Zebra finch but not chicken were then aligned to additional genomes—turkey (*Meleagris gallopavo*), lizard (*Anolis carolinensis*), frog (*Xenopus tropicalis*), Zebrafish (*Danio rerio*), mouse (*Mus musculus*), and human (*Homo sapiens*)—to distinguish songbird novel genes from losses in the galliform lineage (i.e. chicken and turkey).

We found a distinct group of putatively novel or duplicated genes in Zebra finch that do not present an ortholog in Ensembl (e75) and NCBI databases (n = 61). This included several expanded gene sets located on various Zebra finch chromosomes, suggesting that they do not represent local assembly artefacts. We added to this group a set of genes (n = 13) previously reported as expanded in Zebra finch (Supplementary Table Three in [27]), and an additional set of duplicated loci that lack a predictive model (n = 17), detected in the course of BLAT-alignments during the candidate novel gene curation analysis.

To determine whether these genes represented true novel genomic features unique to and shared across songbirds, we conducted BLAST searches of the genomes of 48 avians, including 45 newly sequenced species, representing a broad sampling that covers all major extant radiations of avian diversity (complete list of species and genome assembly in [34, 35]). This provided initial confirmation of the existence of songbird-unique genes, but as the BLAST output was to unannotated scaffolds, putative songbird novel gene sets were analysed in more detail using custom optimized BLAT-alignment algorithm and syntenic analysis in the IGV browser (see Methods) in a subset of these new avian genomes that are of direct comparative interest to our goal of identifying songbird-specific genes: two additional songbirds, the Medium ground finch (*Geospiza fortis*) and the American crow (*Corvus brachyrhynchos*); a suboscine passerine, the Golden-collared manakin (*Manacus vitellinus*); a basal passerine, the Rifleman (*Acanthisitta chloris*); and the Budgerigar (*Melopsittacus undulatus*), a parrot, the sister taxon to Passeriformes. This analysis allowed us to discard spurious hits from the BLAST search. In some cases it also allowed for the identification of the “parent” gene, shown through syntenic conservation to be orthologous between songbirds and non-songbirds, and which underwent duplication to give rise to the novel genes. Confirmed novel genes were categorized into several subsets based on their presumed phylogenetic origin (Figure 1).

We discovered 10 genes that are present in the 3 songbird species studied and absent in all 45 avian non-songbird and 4 non-avian organisms examined, thus representing novel genes that are unique to songbirds. These included 7 cases of gene duplication resulting in a single novel paralog unique to songbirds and 1 case where the gene duplication resulted in two songbird-specific novel paralogs (Table 1; paralogs more generally conserved in passerines or unique to Zebra finch are also included). In some cases the parent gene is known (novel gene names have a terminal ‘L’), in others the parent gene could not be established (gene names have a dash and number). We also identified one entirely novel gene which appears to have arisen *de novo* in songbirds. Altogether, the parent genes and the expanded and *de novo* novel loci comprise a total of 38 genes (Table 1). We describe here their characterization, followed by further details on the general curation effort.

**Table 1:** **Novel genes exclusive to songbirds**

Gene name ^¥^	Phylogeny	Ensembl ID	Gene location	SD site	Brain-derived ESTs	Non-brain ESTs	Non-brain RNA-SEQ
A4GALT-related*							
A4GALT-1	SONGBIRDS^†^	ENSTGUG00000012139	chr1A:65,194,903-65,195,964	YES	No	No	None
A4GALT-2	SONGBIRDS^†^	ENSTGUG00000018227	chr1A:65,202,780-65,203,835	YES	No	No	None
A4GALT-3	SONGBIRDS^†^	ENSTGUG00000018451	chr1A:65,210,658-65,211,719	YES	No	No	None
**CASC1-related**							
CASC1-1	SONGBIRDS^†^	ENSTGUG00000012133	chr1A:65,157,078-65,159,677		FE723736^√^, DV948439	JV165872, JV165873	Embryo, spleen, testes
CASC1-2	SONGBIRDS^†^	ENSTGUG00000012243	chr1A:66,373,778-66,384,478	YES	DV948439	JV184784, JV165872, JV165873	Embryo, liver, muscle, testes
**FN3KRP-related**							
FN3KRP	ALL BIRDS^‡^	ENSTGUG00000007633	chr18:6,574624-6,580,544		DV959265^√^	No	Embryo
FN3KRPL1	SONGBIRDS	No model	chrZ:24,858,422-24,862,943		No	No	Embryo, liver, skin
FN3KRPL2	PASSERINES	ENSTGUG00000006787	chrZ:69,583,022-69,590,574	YES	DV955139^√^, FE727948^√^, DV955139^√^	JV168705√, JV168706√, JR864904√	Embryo, liver, spleen, testes
HYDIN-related*							
HYDIN	ALL BIRDS^‡^	No model	chr11:5,451,491-5,475,997		No	No	Liver, muscle, skin, spleen
HYDINL1	SONGBIRDS	ENSTGUG00000009150	chr11:16,377,691-16,404,553		No	JV172391	Muscle, skin, spleen
HYDINL2	PASSERINES	No model	chr11:16,633,304-16,663,944	YES	No	No	Liver, muscle, testes
HYDINL3	ZEBRA FINCH	ENSTGUG00000009256	chr11:17,005,696-17,033,616	YES	No	No	Muscle, testes
HYDINL4	ZEBRA FINCH	No model	chr11:18,229,584-18,238,051	YES	No	No	None
HYDINL5	ZEBRA FINCH	ENSTGUG00000009737	chr11:19,907,326-19,914,490		No	JV172391	Testes
**NOVEL (TMRA)**							
TMRA	SONGBIRDS	ENSTGUG00000012248	chr1A:66,486,182-66,494,397	YES	CK302958^√^	JV159445√, JV159451√	Embryo, liver, muscle, spleen, testes
**RIOK2-related**							
RIOK2	ALL BIRDS^‡^	ENSTGUG00000001223	chrZ:24,872,816-24,883,105		DV956882^√^	JV172474√, JR863880√	Embryo, liver, muscle, skin, spleen, testes
RIOK2L	SONGBIRDS	No model	chrZ:69,578,247-69,578,886	YES	No	No	Skin
RNF4-related*							
RNF4	ALL BIRDS^‡^	ENSTGUG00000010518	chr4:62,477,216-62,484,738		DV951366^√^	JV183872√, JR867734√	Liver, skin, testes
RNF4L1	SONGBIRDS	No model	chr4:8,201,210-8,201,765		No	No	None
RNF4L2	PASSERINES	ENSTGUG00000018516	chr4:20,660,938-20,751,958		No	No	None
RNF4L3	PASSERINES	ENSTGUG00000018370	chr4:22,411,072-22,433,579		No	No	Embryo, muscle, skin, spleen
RNF4L4	ZEBRA FINCH	ENSTGUG00000018547	chr4:22,445,102-22,478,943		No	No	Testes
RNF4L5	ZEBRA FINCH	ENSTGUG00000018338	chr4:22,507,187-22,517,977		No	No	Muscle, spleen
RNF4L6	ZEBRA FINCH	ENSTGUG00000018226	chr4:22,538,729-22,547,838		No	No	None
RNF4L7	PASSERINES	No model	chr4:41,650,214-41,650,348		No	No	None
**URB1-related**							
URB1	ALL BIRDS^‡^	ENSTGUG00000013442/ no model	chr1:97,555,543-97,592,096/ chr1_random:362,665-365,267	YES	FE722167^√^	No	Embryo, liver, muscle, skin, testes
URB1L1	ZEBRA FINCH	ENSTGUG00000011753	chr1A:60,924,812-60,941,816	YES	CK301434, CK303889, DV957700	No	Embryo, liver
URB1L2	ZEBRA FINCH	No model	chr1A:63,520,638-63,528,208		CK301434, CK303889	No	None
URB1L3	SONGBIRDS	No model	chr5:4,764,796-4,772,823	YES	CK301434, CK303889, DV957700	No	Skin
URB1L4	ZEBRA FINCH	No model	chr7:1,064,071-1,065,024		No	No	None
URB1L5	ZEBRA FINCH	No model	chr23:2,319,460-2,335,606	YES	DV957700	No	Liver, muscle, spleen, testes
YTHDC2-related*							
YTHDC2	ALL BIRDS^‡^	No model	chrZ:21,509,497-21,511,474	YES	No	No	None
YTHDC2L1	SONGBIRDS	ENSTGUG00000014232	chr2_random:378,730-383,125		CK309358^√^	No	None
YTHDC2L2	ZEBRA FINCH	ENSTGUG00000014992	chr3_random:766,156-785,803		No	No	None
YTHDC2L3	PASSERINES	ENSTGUG00000000643	chrZ:10,792,293-10,809,782	YES	No	JV174477, JV177272	Embyro, liver, muscle, spleen
YTHDC2L4	ZEBRA FINCH	No model	chrZ:29,309,810-29,332,222		No	No	Embryo, liver, muscle, skin, spleen, testes
YTHDC2L5	ZEBRA FINCH	ENSTGUG00000003755	chrZ:55,988,800-56,020,229		DV947064^√^	JV174477√	Embryo, liver, muscle, skin, spleen, testes
YTHDC2L6	ZEBRA FINCH	ENSTGUG00000004173	chrZ:57,443,052-57,444,728	YES	No	No	Embryo

Families of genes where at least one member has been determined to be present uniquely in all songbirds. Tandem duplicates, where clear orthology cannot be determined, are distinguished with dashed numbers (e.g., *CASC-1*, *CASC-2*). Other duplicates are named after being ‘like’ their orthologous parent gene (e.g. *RIOK2L*). Ensembl IDs and chromosomal locations refer to the Zebra finch genome. Expressed sequence tags (ESTs) providing evidence of gene expression in the brain [57, 81, 82] and RNA-seq data derived from other tissues [27] are from Zebra finch, non-brain ESTs are derived from Dark-eyed junco [37]. ¥: Gene symbols have been corrected based on our curation of Ensembl annotation, *: Gene family previously reported as being expanded in Zebra finch, ^‡^: Parent gene, ^†^: Parent gene cannot be determined, ^√^: EST is specific to this gene locus.

### Association of novel genes with genomic rearrangement sites

To determine whether the identified novel songbird genes are located close to regions of chromosomal instability in birds, we first generated a map of the sites of avian syntenic disruption (SD) likely representing chromosomal breakpoints, by comparing the syntenic order of Zebra finch vs. chicken genes in SyntenyTracker. We manually verified the consistency of the synteny groups with previous reports [27, 36], using our curated Ensembl models to allow for a more precise identification of genes in the vicinity of the SDs. We also analysed chromosomes 11–28 and Z, which were not included in previous studies (Additional file 1: Table S6). Finally, we examined the syntenic arrangement of these blocks in several outgroups (lizard, mouse, human) to distinguish SDs that are specific to the chicken lineage, where the flanking regions of Zebra finch blocks are either identical or highly similar to those in non-avian species (highlighted in pink in Additional file 1: Table S6), from SDs specific to the songbird lineage, where the flanking regions of Zebra finch blocks differ from that of the other species examined (highlighted in blue-green in Additional file 1: Table S6). Compared to previous studies [27, 36], this analysis resulted in a refined and comprehensive list of SDs representing likely chromosomal breakpoints specific to the songbird lineage.

**Figure 2:**
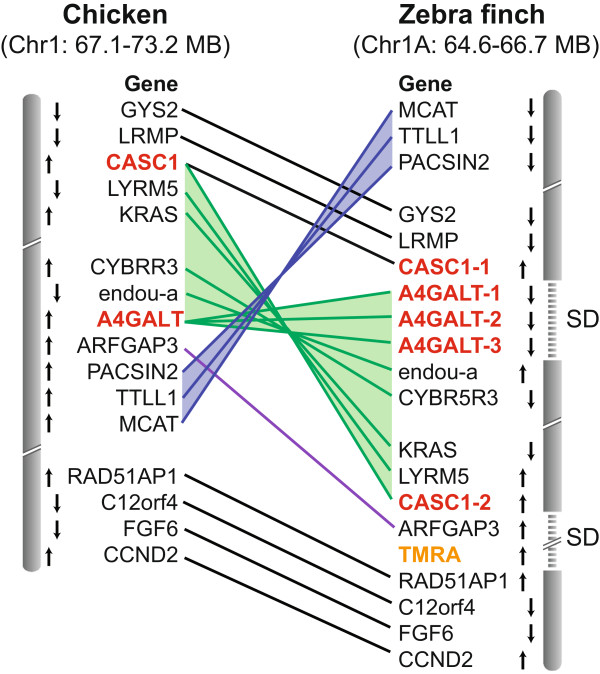
**Example of songbird novel and duplicated genes associated with regions of syntenic disruption.** Chromosomal maps of the syntenic order of genes in chicken (chr1) compared to Zebra finch (chr1A) reveal that songbird-unique *de novo* novel gene (*TMRA*, in orange) and duplicated/expanded novel genes (*CASC1*, *A4GALT*; in red) are located in chromosomal regions containing syntenic disruptions (SD) that are unique to the songbird lineage (i.e. the syntenic flanking genes in Zebra finch are different from those in other avian and non-avian species). Small black up/down arrows next to each gene indicate orientation on the minus/plus strand of DNA. Line colours denote genes in rearranged syntenic blocks, with shaded regions representing apparent chromosomal inversions.

Next, we examined whether novel songbird genes as well as their parent genes map within or near songbird lineage-specific SD sites. We found that 6 of 10 novel genes are present at the start, end, or within SD regions (Table 1, position of SDs indicated in Additional file 1: Table S6). This suggests that a large proportion of the duplication events that gave rise to novel genes seems to have occurred in regions of chromosomal instability. Two regions were associated with multiple duplication events: a chromosomal inversion in chr1A (expansions of *A4GALT* and *CASC1*, and occurrence of *TMRA*; Figure 2), and a chromosomal rearrangement on chrZ (associated with the expansions of *FN3KRP* and *RIOK2*). Interestingly, most of the putative *de novo* novel (*n* = 6 of 8) and ~50% of the duplicated (*n* = 18 of 32) genes that are not unique to songbirds (found in subsets of songbirds, all passerines, or other Neoaves; Additional file 1: Table S5) are also located within or close to SDs, supporting the association between chromosomal breakpoints and the emergence of novel genomic features in non-songbirds as well.

### Predicted protein analysis of songbird-unique genes

We assessed the potential function of novel songbird genes by examining the predicted protein domains of the largest open reading frame. These were obtained either from the Zebra finch Ensembl model, or by mapping the Zebra finch parent gene and its chicken ortholog onto the novel gene locus, in cases where an Ensembl model for the novel duplicate was absent or incomplete (in the latter case noting the possible occurrence of additional unmapped exons/domains in nearby regions). We then compared the predicted domains between parent and duplicate genes to identify potential changes in function. As an outgroup to birds, we verified the domain organization of the parent genes in humans, where gene predictions are often more complete than in other vertebrates.

In one case, we found one gene where the duplicate copies are nearly identical. The *A4GALT* expansion consists of 3 complete tandem copies; all contain a glycosyltransferase DXD-sugar binding motif and an alpha 1,4-glycosyltransferase domain, the latter involved in protein glycosylation. Due to high conservation, it is hard to determine which copy is orthologous to the ancestral *A4GALT*. For the remaining gene families, we found differences in domain annotation between the duplicate and parent genes, with some genes representing partial copies and others gaining additional exons.

For *RIOK2*, the parent gene contains a RIO-like kinase, a RIO2 kinase N-terminal domain, a winged helix domain that confers DNA binding properties, and a coiled-coil domain, indicative of a function in the regulation of gene expression. The model-less *RIOK2L* contains only the coiled-coil domain, and thus is a partial duplicate. Since there are no sequence gaps upstream of the model, the missing N-terminal domains and exons cannot be in a gap. Interestingly, *RIOK2L* overlaps with, and is antisense to, one of the 3’-splice variants of *FN3KRPL2*.

As in the chicken and human orthologs, *FN3KRP* and its duplicate paralog present in all passerines (*FN3KRPL2*) contain six exons, and the predicted large fructosamine-3-kinase domain that covers most of the open reading frame is indicative of a role in deglycation and functional activation of proteins protective against hyperglycemia. *FN3KRPL2* has additional downstream exons, part of several transcripts that are alternatively spliced in the 3’UTR region of this gene. The copy specific to songbirds (*FN3KRPL1*), however, lacks the amino-terminus exon (~45 aa residues) of the parent gene. Since there are no sequence gaps upstream of this locus where that exon might be located, this copy is partial, with a likely disruption of the main functional domain.

Both copies of *CASC1* in songbirds lack a coiled-coil domain close to the amino-terminus present in the chicken and human orthologs. However, since the predicted peptides lack a starting codon, this domain may be present in the gaps upstream to the models. Furthermore, *CASC1-1* is shorter than *CASC1-2*, consisting only of the 282 amino acid residues at the carboxy-terminus.

As in chicken and human, the parent *URB1* gene in songbirds contains a predicted nucleolar pre-ribosomal-associated protein 1 domain close to the amino-terminus and an armadillo (ARM-) like fold involved in interactions with other proteins and nuclei acids. The expanded set of duplicate *URB1* copies do not contain either of the domains above, although in some cases the missing domains might be hiding in gaps.

**Figure 3:**
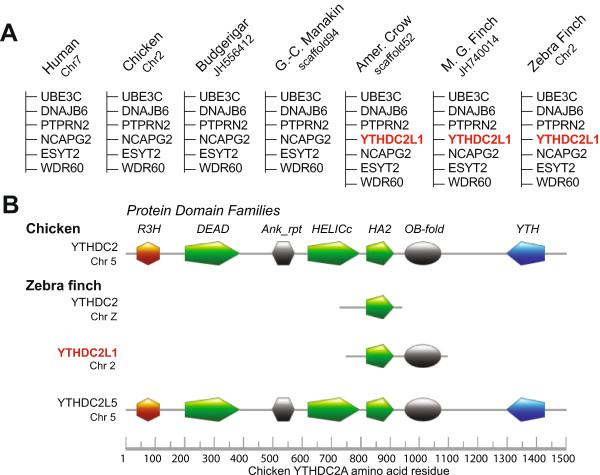
**Syntenic and protein functional domain analysis of** ***YTHDC2L1*** **and** ***YTHDC2L5.*** **(A)** Schematic representation of conserved chromosomal loci in avian and non-avian vertebrate species showing the relative position of *YTHDC2L1* (in red), a novel expansion of *YTHDC2* that is only present in songbirds. Adjacent genes are indicated in black. The chromosome or scaffold number is indicated beneath each species common name. **(B)** An alignment of the protein family domains predicted for amino acid sequences derived from Chicken *YTHDC2*, the orthologous *YTHDC2* “parent” gene in Zebra finch, as well as the copies of *YTHDC2* that are only present in songbirds (*YTHDC2L1*), and Zebra finch (*YTHDC2L5*). Specific protein family domains predicted by InterProScan5 are aligned relative to Chicken *YTHDC2*, and are indicated by the various coloured symbols. *YTHDC2* and *YTHDC2L1* lack nearly all of the major protein family domains that are characteristic of *YTHDC2*. In contrast, *YTHDC2L5* appears to be a nearly complete copy of *YTHDC2*.

In the case of the *YTHDC2* expansion, the expanded set includes several copies that are differently shared across species, and which display marked changes in the predicted structure across the different copies (Figure 3A). In human and chicken, the parent gene contains several domains (R3H, DEAD, Ank_rpt, HELICc, HA2, OB-fold, YTH) involved in functions like binding to and inducing conformational changes in single stranded nucleic acids (RNA or ssDNA). In songbirds it is highly truncated (*YTHDC2*, Figure 3B), lacking most predicted domains. The songbird-unique duplication (*YTHDC2L1*, Figure 3B) also lacks all domains except the HA2 and OB-fold. The alignment of an EST containing a polyA at this locus confirms that the sequence is complete at the 3’ end, thus this copy lacks the YTH domain. All 3 songbirds contain relatively complete copies of the gene that lack the amino-terminus (~74 residues) but contain most other domains of the parent gene at different syntenic locations (e.g. *YTHDC2L5* in Zebra finch, Figure 3B)*.* Overall, these observations suggest continued expansion of this gene following divergence of songbirds.

All *HYDIN*-related copies are partial compared to the parent gene in chicken and human, which contains a predicted PapD-like domain related to periplasmic chaperoning function, and a P-loop NTPase domain thought to be involved in modulating conformational changes in other proteins. The Zebra finch *HYDIN* parent gene contains only a short fragment of the parent gene and has no predicted model; all other duplicates contain truncated portions of the 5’ part of the parent gene. Because these loci are flanked by gaps and several unassembled sequences (chrUn) contain portions of the gene, some of which close to the 3’ end, several assembled loci may be partial due to assembly incompleteness. We also note that the alignment scores of the chicken parent gene and songbird-expanded copies are rather low, reflecting considerable divergence.

Similarly to the orthologous gene in chicken and humans, *RNF4* and its duplicates in passerines each contain a predicted single RING finger motif close to the carboxy-terminus. However, the songbird-specific copy and one of the copies shared by passerines are truncated, lacking most of the 5’ half of the gene. Low alignment scores of these duplicates point to significant divergence from the parent locus.

**Figure 4:**
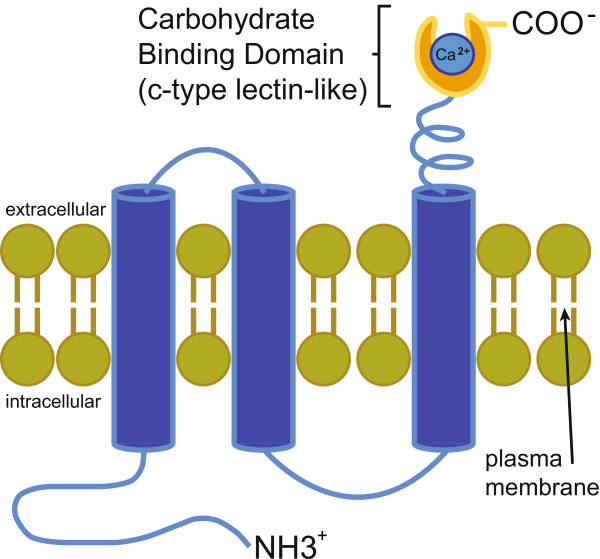
**Schematic model depicting the predicted structure of** ***TMRA*** **in the plasma membrane.** Protein functional domain analysis (InterProScan5) predicts the protein coding sequence of *TMRA* contains three transmembrane spanning domains connected to an extracellular C-type Lectin-like (CLEC) domain that is typically associated with carbohydrate binding.

We identified one novel gene (ENSTGUG00000012248) with no identifiable parent gene that appears to have arisen *de novo* in the songbird lineage. Its only trace outside of songbirds is in the form of a short, truncated segment of one of its coding exons in the correct syntenic position in two non-songbird species (Peregrine falcon and Bar-tailed trogon, data not shown). Its predicted protein (330 aa) contains a putative amino-terminus cytoplasmic domain, three transmembrane domains, and a carboxy-terminus extracellular domain, the latter with a putative C-type lectin domain (Figure 4). Thus, it appears to encode a polytopic transmembrane α-helical protein, suggesting a role related to the cell surface, possibly involving carbohydrate binding activity. Due to a gap in the Zebra finch genomic sequence, this analysis required sequencing an ESTIMA cDNA, CK302958, the longer of two brain-derived cDNAs that map specifically to this locus (complete sequence submitted to GenBank, accession ID:KM520127). Results were confirmed by comparing cloned cDNAs from the Dark-eyed junco, another songbird species [37]. Based on its predicted structure and discrete expression in the song nucleus RA (see below), we annotated this gene *TMRA* (transmembrane protein of the robust nucleus of the arcopallium).

### Expression analysis of songbird-unique genes

To gain insights into the possible functional roles of the identified novel songbird genes, we performed an expression analysis using both publicly available expressed sequence tags (ESTs) and RNA-seq transcription data [27, 37] from brain, liver, muscle, skin, spleen, testes, and whole embryo of Zebra finch and Dark-eyed junco; as well as *in situ* hybridization of Zebra finch brain sections. Our results fell into several categories: songbird novel genes with no evidence of expression in these tissues (*A4GALT*, *RNF4L1*), expressed in non-brain tissues only (*FN3KRPL1*, *HYDINL1*, *RIOK2L*), expressed in multiple tissues including brain (*CASC1*, *TMRA*, *URB1L3*), and one gene with expression detected solely in the brain (*YTHDC2L1*). In the case of *A4GALT*, we found no evidence for expression of either the parent gene or the duplicate copy unique to songbirds in these tissues. In contrast, for all other novel genes, we find that transcriptional data reveal differential tissue expression of parent and novel genes, suggesting functional differentiation among loci (Table 1).

In some cases, songbird novel genes show more limited expression than their parent genes. Songbird-unique gene *HYDINL1* has lost expression in liver relative to parent gene *HYDIN*. For the *RIOK2* and *RNF4* gene expansions, we found evidence of expression in brain and other tissues for the parent genes, but limited (*RIOK2L*, expressed only in skin) or no expression (*RNF4L1*) of the songbird duplications (Table 1). In both cases, we were unable to detect parent gene expression in the brain by *in situ* with probes from brain-derived cDNAs, suggesting that expression levels are either very low and/or brain state-dependent.

In other instances, the songbird duplication was expressed in additional tissues beyond those of the parent gene, as in the case of *FN3KRPL1*, which is expressed in liver and skin, as well as sharing expression in embryo with parent gene *FN3KRP* (Table 1). Several brain-derived ESTs map specifically to parent gene *FN3KRP* and to passerine duplicate *FN3KRPL2*, thus both loci are transcriptionally active in the brain. Interestingly, several transcript variants for *FN3KRPL2* differ on the length of the 3’UTR region. *In situ* hybridization revealed that *FN3KRP* and *FN3KRPL2* expression, including several transcript variants for the latter, is uniformly low throughout the brain, including nuclei of the song system (data not shown). In contrast, there was no evidence that songbird-specific *FN3KRPL1* is transcriptionally active in the brain.

**Figure 5:**
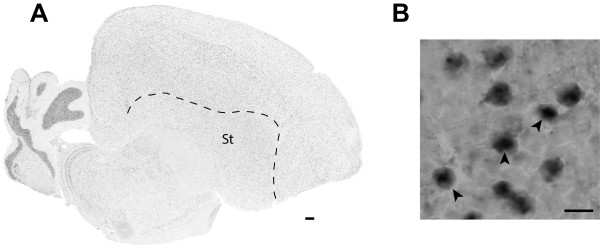
**Expression of** ***URB1*** **in the adult male Zebra finch brain. (A)** Photomicrograph of *in situ* hybridization showing uniform expression of *URB1* in the pallium. **(B)** High-power view reveals *URB1* enrichment in individual pallial neurons. Note that in several of the cells indicated by the arrowheads the intracellular labelling appears more robust in the nucleus than in the surrounding cytoplasm, forming a pattern reminiscent of a fried-egg. Scale bar: 10 μm.

With *URB1*, we found further evidence of expressional divergence. RNA-seq showed the parent gene to be expressed widely in embryo, liver, muscle, skin, and testes; whereas expression of songbird duplicate *URB1L3* was only detected in skin (Table 1). *In situ* hybridization revealed uniform brain expression of the parent gene and duplicate copies (Figure 5A). However, due to cross-alignment of probes, we cannot unequivocally assign cDNAs from the duplicate copies to a specific locus. At higher resolution, the labelling of both parent and duplicate genes is cellular, but rather than displaying the cytoplasmic pattern typical of most mRNAs, expression is concentrated within nuclei, consistent with nucleolar localization (Figure 5B).

**Figure 6:**
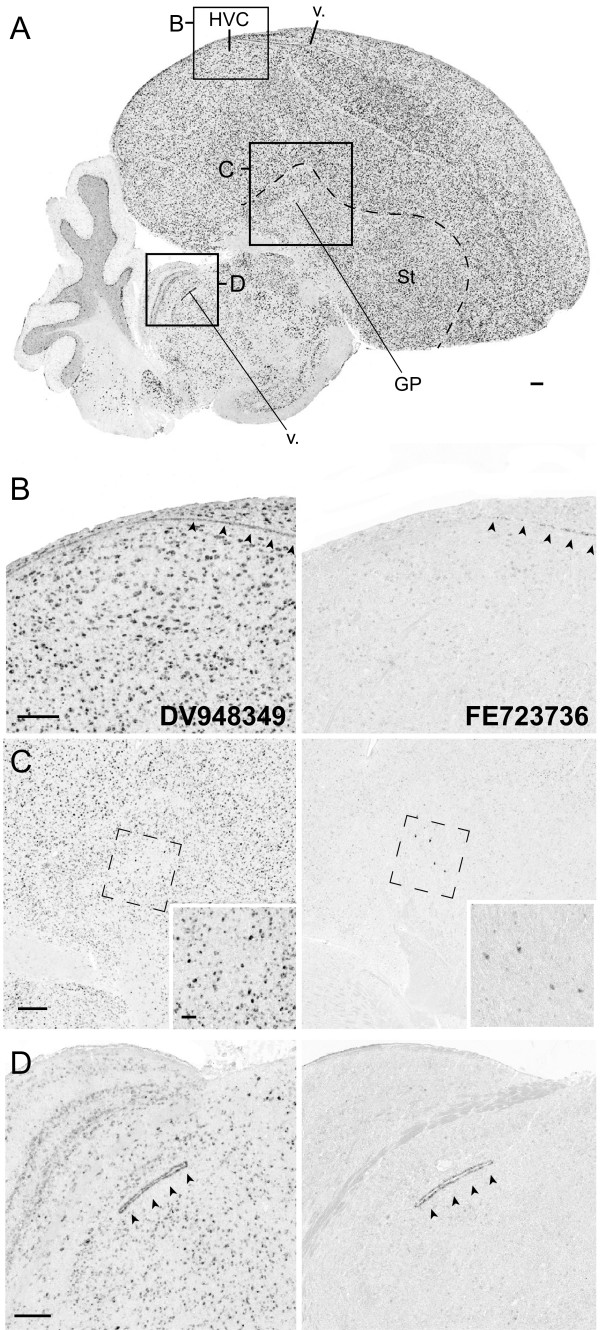
**Differential expression of** ***CASC1*** **genes in song nucleus LMAN of the adult male Zebra finch. (A)** Photomicrograph of an *in situ* hybridization conducted with a probe (DV948439) that is not locus specific reveals expression of *CASC1-1* and/or *CASC1-2* throughout the brain (See Additional file 1: Figure S3 for details). The approximate positions for the photomicrographs shown in panels B-D are depicted by the black squares. **(B-D)** *CASC1-1/2* mRNA is highly expressed in song nucleus HVC (**B**, left panel), the globus pallidus (**C**, left), and ependymal cells of the lateral ventricle (**D**, left; arrowheads). In contrast, *CASC1-1*, revealed by a probe that is specific to this locus, is differentially expressed in ependymal cells of the lateral ventricle (**B**, right panel), large likely GABAergic cells in globus pallidus (**C**; right), and ependymal cells of the fourth ventricle in the midbrain (**D**, right). The dashed rectangles in B indicate the approximate positions of the high-power photomicrographs depicting labelled cells in the globus pallidus. Anatomical abbreviations: HVC, proper name; GP, Globus pallidus; St, striatum; v., lateral ventricle. Scale bars: 100 μm.

In the case of *CASC1-1* and *CASC1-2*, RNA-seq revealed shared expression in embryo and testes, with *CASC1-1* also expressed in spleen and *CASC1-2* detected in liver and muscle (Table 1). We identified two Zebra finch brain cDNA clones, FE723736 and DV948439, associated with *CASC1-1* and *CASC1-2* (Additional file 2: Figure S1). FE723736 aligns completely (98.1% identity) to exons 2–5 of the 5-exon *CASC1-1*. It also aligns well (98.9% identity) to *CASC1-2*, but this alignment is partial, since the *CASC1-2* locus lacks the 100 bp 4^th^ exon predicted in *CASC1-1* and present in FE723736. Thus, FE723736 is transcribed from *CASC1-1*, indicating that this gene is unequivocally expressed in the brain. In contrast, Zebra finch clone DV948439 aligns completely with high scores to both loci, thus we cannot establish from which locus it is transcribed. Because we cannot exclude the possibility that both clones are transcript variants from *CASC1-1*, we cannot conclusively establish whether *CASC1-2* is transcriptionally active in the brain. Since we use high stringency hybridization conditions, and these clones are relatively small, we predicted that we might detect differences in their brain distributions, if present. Indeed, these clones displayed partially overlapping but distinct patterns. DV948439 revealed strong labelling throughout the brain, including the pallium, thalamus, and both granular and Purkinje cell layers of the cerebellum (Figure 6A). The distribution and density of labelled cells in the pallium was uniform, but the relative level of expression varied from cell to cell (Figure 6B,C, left panels). Fiber tracts and white matter were devoid of signal, suggesting the probe is detecting transcripts that are not expressed in glia, but cells in walls of the lateral ventricles were strongly labelled (Figure 6D, left panel). In contrast, FE723736 revealed specific expression in the cells that define the ventricular wall (Figure 6B,D, right panels), and in large neurons within the globus pallidus (Figure 6C, right panel).

**Figure 7:**
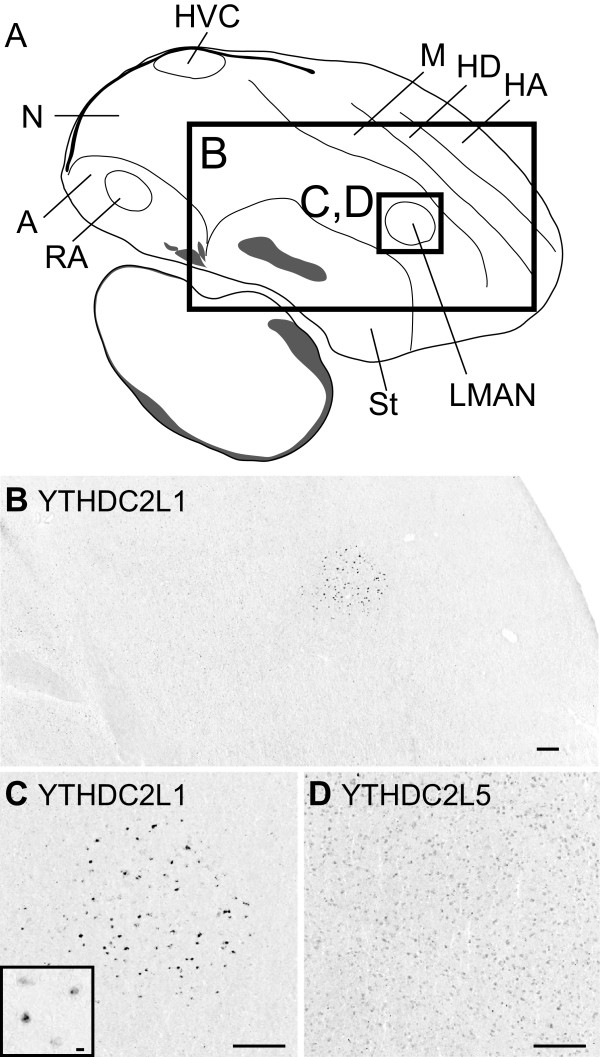
**Differential expression of** ***YTHDC2L1*** **in song nucleus LMAN of the adult male Zebra finch brain. (A)** A schematic depicting a sagittal brain section ~2 mm from the midline shows the approximate location of the *in situ* photomicrographs presented in panel B. **(B)** Photomicrograph of *in situ* hybridization of songbird novel gene YTHDC2L1 shows discrete expression in song nucleus LMAN. **(C)** Detailed view of this section reveals that expression of YTHDC2L1 is restricted to large cells of LMAN, with labelled foci evident in some cells pairs within cellular nuclei (inset). **(D)** A comparable view of paralogous gene YTHDC2L5 shows low levels of expression, non-differential in LMAN. Anatomical abbreviations: A, arcopallium; H, hyperpallium; HVC, proper name; LMAN, lateral magnocellular nucleus of the nidopallium; M, mesopallium; MD, dorsal mesopallium; MV, ventral mesopallium; N, nidopallium; RA, robust nucleus of the arcopallium; St, striatum. Scale bars: 100 μm in B–D; 20 μm in C inset.

The truncated *YTHDC2* parent gene lacks any EST or RNA-seq evidence of expression, supporting the conclusion that it is functionally inactive in songbirds (Table 1). We identified several Zebra finch brain cDNA clones that align to varying degrees to the 7 *YTHDC2*-related genes (Additional file 2: Figure S2A). Based on alignment scores and the presence of unique exons, we were able to unequivocally assign two clones to specific copies of *YTHDC2* (Additional file 2: Figure S2B). CK309358 aligns with 99.9% identity to songbird-unique *YTHDC2L1*, including a 3’ exon that is not present in any of the other *YTHDC2*-related loci, whereas DV946054 aligns with 100% identity to Zebra finch-unique copy *YTHDC2L5*, including a first exon that is not present at any other loci. Thus, both *YTHDC2L1* and *YTHDC2L5* are transcriptionally active. *In situ* hybridization reveals that *YTHDC2L5* is expressed at low levels throughout the brain (Figure 7D), with RNA-seq data revealing expression in all other tissues examined (Table 1). In contrast, songbird copy *YTHDC2L1* appears to be exclusively expressed in LMAN (Figure 7B,C), a brain nucleus critical for song learning and vocal variability [38, 39]. Strongly labelled cells are uniformly distributed throughout LMAN, displaying cellular labelling that is diffuse in the cytoplasm and strong in the nucleus. The labelled cells have very large somata (>17 μm; Figure 3C, inset), indicating that they likely correspond to the large magnocellular projections neurons that are found within the core region of LMAN [40]. In some cases we note that we also detect signal within the nucleus in the form of labelled foci, suggesting that we may be detecting sites of active transcription (Figure 7C, inset).

**Figure 8:**
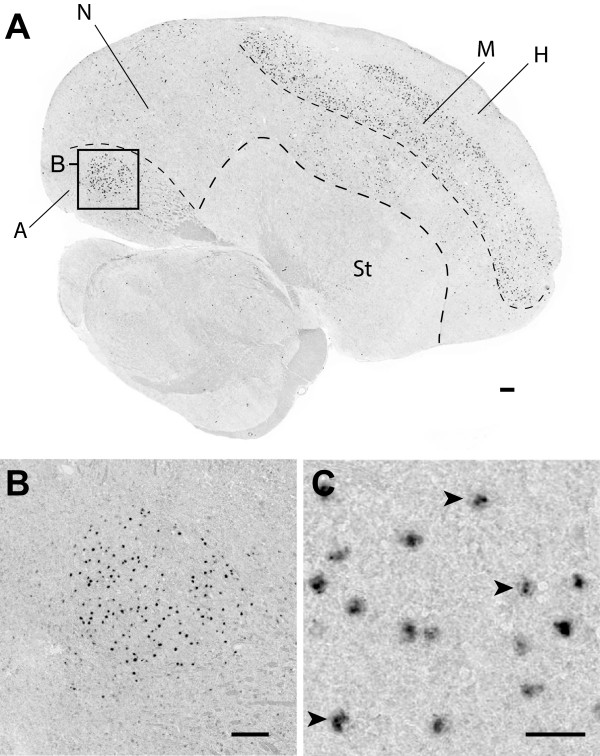
**Differential expression of** ***TMRA*** **in song nucleus RA of the adult male Zebra finch. (A)** Photomicrograph of *in situ* hybridization showing the distribution of *TMRA* expressing cells in a parasagittal brain section that includes song nucleus RA (~2.0 mm from the midline). **(B)** High-power view reveals the enrichment of *TMRA* in individual cells within RA. **(C)** Detailed views reveal that in some cells in RA (indicated by the arrowheads), labelling is largely restricted to nuclei, and expression is low in the surrounding cytoplasm. Anatomical abbreviations: A, arcopallium; H, hyperpallium; M, mesopallium; N, nidopallium; St, striatum. Scale bars: 100 μm in A and B; 20 μm in C.

For the *de novo* gene *TMRA*, RNA-seq data detected wide expression in embryo, liver, muscle, spleen, and testes; brain expression was shown by the presence of finch and junco cDNA clones which mapped specifically to this locus (Table 1). *TMRA* is sparsely expressed throughout the nidopallium, but enriched in the mesopallium (Figure 8A; mesopallium as recently defined in [41, 42]). Most strikingly, *TMRA* is a prominent marker of song nucleus RA (Figure 8A,B), a structure required for the production of learned song [16, 43]. Labelled cells have large somata (Figure 8C), a characteristic feature of established populations of HVC and RA projection neurons [44, 45]. As with *URB1* and *YTHDC2*, expression is enriched in cellular nuclei (Figure 8C; arrowheads), and in many cells labelling appears focal, perhaps indicating that the transcript is concentrated in nucleoli, or that we are detecting independent transcriptional sites.

### Curating misannotated novel genes in songbirds

Many genes in the candidate set, including those reported in previous studies (Supplementary Table Three in [27]), did not pass our criteria for songbird-unique novel genes. We provide corrected annotations for these false positives, based on the results of the comparative BLAT alignments and syntenic analysis, subdividing them into several categories (Additional file 1: Tables S1, S2, S3, S4 and S5).

A large set of apparent duplications consisted of typically large, multi-exonic genes where the Ensembl prediction failed to group all exons under the same model, resulting in two or more partial adjacent models annotated as duplicates or expansions of that ortholog (n = 203; Table S1). Such cases were easily identified by examining the BLAT alignment to the Zebra finch genome of a more complete ortholog from a different species such as chicken, mouse, or human (for example, Additional file 2: Figure S3A).

Another large group consisted of separate gene pairs or sets, often members of the same gene family, where one or more models were misidentified as a gene duplication or expansion. These false positive duplications were found in tandem on the same chromosome (n = 90, Additional file 1: Table S2A; for example, Additional file 2: Figure S3B), or on separate chromosomes (n = 59, Additional file 1: Table S3A). In other cases, the models represent true duplicate pairs, but these expansions are also present in chicken and/or non-avian organisms, and thus they are not unique to songbirds (n = 97 adjacent models, Table S2B; and n = 68 models from separate chromosomes, Additional file 1: Table S3B). Some of these are also present in lizard, frog, and/or fish, but not in mouse or human, thus they appear to be duplications that occurred in a distant vertebrate ancestor but were lost in mammals (e.g. *MRC1-1* and *MRC1-2*). Other cases appear to have originated in birds, as they also occur in chicken and/or turkey, but not in non-avian species (e.g. *TTR-1* and *TTR-2*).

Among Zebra finch genes misannotated as novel and without identified orthologs in other species, the vast majority consisted of short segments of known genes that were not incorporated into the main Ensembl model, likely due to sequence gaps or regions of low assembly quality (n = 154, Additional file 1: Table S4A). A smaller subset consisted of previously uncharacterized genes, described as novel by Ensembl, but orthologous loci could be identified in chicken and/or other organisms (n = 64, Additional file 1: Table S4B). Of note, some of the chicken loci in this subgroup did not have a predictive Ensembl model but could be found at the correct syntenic location through BLAT-alignment.

A small set of candidate novel Ensembl gene models (n = 25) were found to be part of massively expanded gene families (e.g. olfactory receptors, keratins, zinc fingers). As their curation would require extensive further analysis to establish correct orthology, these were not studied further. Finally, a subset of candidate novel genes (n = 28) displayed a loss of intronic regions and had flanking repetitive elements (e.g. LTRs, LINEs, SINEs) characteristic of retrotransposon-mediated duplication. These genes will be further reported elsewhere.

Our phylogenetic searches revealed some gene duplications present in Zebra finches but in no other species (n = 32). While these could represent real Zebra finch-specific features, in most cases the two genes in a duplicated pair occur in tandem, often in regions of poor sequence quality, flanked by gaps, and tend to have very high (>95) percent identities. They likely represent assembly artefacts, due to improper placement of allelic variants or misassembly of repetitive sequences. We are currently generating further sequence and a new genome assembly for the Zebra finch, and intend to further examine this subset elsewhere.

A distinct subset of novel Zebra finch genes were also present in the Medium ground finch but not in the crow or in any other bird; they thus appear to be specific to the finch lineage (n = 17, Additional file 1: Table S5A). In other cases, the genes were present in all passerines (3 songbirds, manakin, and Rifleman) but in no other birds; they thus represent features shared among Passeriformes (n = 16, Additional file 1: Table S5B). Yet other cases could be found in numerous avian species, but not chicken; these likely originated early in the radiation of Neoaves, or represent genetic losses specific to Galliformes (n = 7, Additional file 1: Table S5C).

## Discussion

Our analysis identifies with high certainty several songbird-unique genes, and drastically reduces the number of genes misannotated as novel by automated detection algorithms [46, 47]. In addition to significantly extending and improving upon previous lists of songbird novel genes [27, 31], this effort demonstrates the necessity of a systematic curation pipeline that incorporated synteny analysis in order to accurately predict gene identity, and establishes a template for using comparative genomics to identify novel genes in any genome. The pipeline is particularly effective in eliminating false positive novel gene annotations by identifying orthologs undetected by automated gene prediction algorithms due to gene model incompleteness. This effort also illustrates how analysis of a large number of genomes can enable the discovery of genomic features unique to specific groups and possibly associated with group-specific traits, a strategy that will become increasingly feasible as larger collections of genomes from other animal groups become available.

Although we have focused on the contribution of novel genes to the evolution of the songbird lineage in this study; other factors are likely to have played a role as well; including differential gene substitution rates, chromosomal rearrangements, retrotransposon-related events, and modification of regulatory regions. Several of these are being explored in companion papers to this study [34, 35].

Lineage-specific expansions have been reported in Zebra finch versus chicken [27], but the incorporation of 45 newly sequenced, high-coverage (30–120X) avian genomes as well as representative non-avian genomes allows us to identify the specific set of duplications that arose following the divergence of ancestral oscine passerines from their closest living relatives (i.e. suboscine passerines, ~32 mya), but before the songbird crown radiation which includes both finches and crows (~20 mya) [34]. We note that this represents a high-confidence set: if these genes were not unique to songbirds, and their absence in non-songbirds a consequence of incomplete sequencing, we would expect them to be randomly distributed across the 45 non-songbird genomes sequenced. Instead, we find them only in songbirds, and in none of the non-songbird species examined. Due to our strict criteria, our list of songbird-unique genes is likely an underestimate, as we have excluded loci not mapped to a known chromosome, due to the possibility that these represent alleles rather than actual paralogs, as well as gene sets annotated by Ensembl as one-to-many orthologs, which require further analysis to establish exact orthology. In addition to identifying genes uniquely present and shared among songbirds, this analysis also reveals further sets of lineage-specific genes which characterize finches, passerines, neoavian birds, or Galliformes. The identification of gene sets common to these avian clades represents a significant advance for identifying genomic innovations whose emergence may be linked to some of the characteristic traits of these groups.

Our identification of SDs that emerged following evolutionary divergence of Galliformes (e.g. chicken) and Neoaves (e.g. Zebra finch) substantially improves on previous studies [27, 36] by refining the location of SD sites, identifying breakpoints on chrs 11–28 and Z, and distinguishing SDs present in Zebra finch only, thus possibly specific to songbirds, from those present in chicken only, and thus possibly specific to Galliformes. The fact that the majority of novel genes, both those unique to songbirds as well as those present in other avian groups, are located within or immediately adjacent to SDs suggests that chromosomal rearrangement is a major mechanism for the emergence of novel genomic features in passerines and other avian groups, as found in other lineages [48, 49]. This corroborates previous reports establishing non-allelic homologous recombination following inter- or intra-chromosomal rearrangement as an essential mechanism for genome evolution [50, 51]. Of note, songbird chromosomes 1 and 1A are known to have undergone significant rearrangement, having split from chicken chromosome 1 [52], which our findings suggest has had repercussions for novel gene evolution: a single rearrangement on Chr1A can be associated with multiple novel genes; another SD on Chr1 harbours the previously described growth hormone gene duplication (*GHL*) [53], which we conclude to be passerine-specific. Related to these rearrangements, avian SDs have been previously associated with a high occurrence of repetitive elements [36], which are thought to provide a substrate for non-allelic homologous recombination and genomic instability [54, 55]. It is also possible that some of the genes reported here, as well as others containing repetitive elements, may have arisen through retrotransposon-mediated duplication mechanisms [56].

A major outcome of this study was the discovery that most of the novel songbird genes are transcriptionally active, with both EST and RNA-seq data supporting differential tissue expression of parent genes and songbird novel loci, indicating a diversification of function following duplication. Compellingly, some novel genes’ brain expression indicates an association with songbirds’ neural system for learned vocalizations, suggesting that their evolution could be related to the emergence of this characteristic songbird trait. Although other studies have identified gene enrichments in song nuclei [57–59], this is the first report that genes found only within the songbird lineage are transcriptionally active in these nuclei. This suggests that certain novel genomic features of songbirds may have evolved to support the function of the circuitry dedicated to vocal learning behavior. For other novel genes, transcriptional evidence suggests that their function is associated with other, non-neuronal tissues, including skin, muscle, liver, and testis. Although more targeted experimental approaches involving gene manipulations will be required to establish the exact functions of songbird novel genes, we discuss potential implications in the context of their predicted protein domain architecture and selective tissue expression.

The multiple copies of *A4GALT* are complete, thus this gene expansion might represent an increase in molecular function, or a diversity of functions if accompanied by divergent expression patterns. Although we found no evidence of expression of these genes, in other organisms *A4GALT* has been implicated in glycosylation of surface antigens related to the P blood group system, indicating that this gene and its expansion are likely related to organs and systems outside of the range of tissues explored in our analyses [60].

Several other gene expansions (*RIOK2*, *RNF4*, *URB1*, *HYDIN*) are predicted to encode much shorter proteins that lack specific domains compared to the parent genes, sometimes even lacking a recognizable ORF. For these genes, there are indications of differential expression in non-brain tissues by RNA-seq, although we cannot unequivocally demonstrate brain expression due to cross-alignment of cDNAs to multiple loci (Table 1). These truncated genes could act as partial competitive inhibitors of the parent gene, as seen with the human specific duplication of the *SRGAP2* gene, which in turn causes slower brain development in humans relative to other mammals [29]. It is also possible that these might represent pseudogenes resulting from a complete duplication followed by a degradation of the coding sequence and loss of transcriptional activity of one paralog. A notable exception was *FN3KRP*, a gene related to deglycation of proteins and thus possibly protective against hyperglycemia [61]. *FN3KRPL2*, which is shared by all passerines and complete in terms of coding domains, has even gained complexity in the form of multiple 3’UTR variants. We also note that in some cases we found evidence of brain expression of the parent gene only (e.g. *RIOK2* and *RNF4*, both low to undetectable by *in situ* but with associated cDNAs) or of the parent gene and its duplicates (*FN3KRP*, *URB1*). While the patterns were broad and uniform, thus uninformative with regards to regional specializations, they establish a link to basic, non-specialized aspects of brain function. Intriguingly, we note that two songbird novel genes exhibit exclusive expression in skin (*RIOK2L*, *URB1L3*), a finding without clear precedent that points to potential unexplored molecular specializations of songbirds.

There is significant sequence divergence between *CASC1-1* and *CASC1-2*, which, along with the differential expression patterns detected, suggests a divergence of molecular function. The restricted expression of the *CASC1-1* in the ventricular zone is intriguing, given that the parent gene is related to the control of cell proliferation [62], and the subventricular region adjacent to the ventricles is a site of continued proliferation of neuronal precursor cells in adulthood [63, 64]. The other transcript is broadly expressed but cannot be unambiguously linked to either paralog. One possible interpretation is that the two paralogs have very distinct expression patterns. In this regard, the chromosomal rearrangement that gave rise to this duplication (Figure 8) likely disrupted the regulatory promoter of *CASC1-1*, leading to differences in expression patterns. Alternatively, the two transcripts analysed might be variants of *CASC1-1*, with *CASC1-2* representing a pseudogene. In either scenario, further studies of the *CASC1* duplication are worth pursuing, and analysis of additional genes in the proximity of genomic rearrangement sites could lead to further insights into evolving patterns of gene regulation in the avian brain.

The *YTHDC2* gene expansion clearly illustrates a divergence of function across paralogs, which differ in both structure and expression. *YTHDC2* is predicted to encode a protein capable of binding to RNA (through its YTH domain [65]) and inducing conformational changes (through its RNA helicase activity [66]). Although the functionally complete copy *YTHDC2L5* is expressed broadly in the brain and other tissues, the songbird-unique copy *YTHDC2L1* is expressed solely in song learning nucleus LMAN in the brain, and is highly truncated relative to the parent gene, retaining only the HA2 and OB-fold domains associated with RNA helicase activity regulation [67]. This indicates a neofunctionalization of songbird paralog *YTHDC2L1*, and suggests that it may play a role in RNA regulation in LMAN, a conclusion that will await targeted experimental confirmation.

It is unclear how the complete *TMRA* arose in songbirds, but given the presence of a short, exonic segment in the correct syntenic position in falcon and trogon, two clades recently shown to be closely related to songbirds [34], *TMRA* appears to represent a *de novo* gene gain in songbirds with ancestral non-coding origins, as shown previously for genes which originated *de novo* in human from non-coding sequences in chimp [68]. *TMRA* is a remarkable marker of song nucleus RA, suggesting a role related to the neural coding of learned vocalizations, as RA represents the cortical output for vocal-motor control and is essential for the production of learned vocalizations [16, 43]. *TMRA* is a member of the *CLEC* family of transmembrane protein genes, with a function likely associated with cell surface recognition processes required for cell-cell and/or cell-substrate interactions [69]. Although one cannot exclude the possibility that *TMRA* may play a role in response to pathogens, as occurs for other members of the lectin family [70], we note that members of some gene superfamilies related to immune system function (e.g., *N-CAM*) also play major roles in the nervous system, modulating cell-cell adhesion and interactions with extracellular matrix that are critical for neural development and function [71].

The discovery of novel songbird genes expressed specifically in the vocal control system provides evidence that some molecular specializations unique to this group may be associated with vocal learning. This trait evolved in three avian lineages (songbirds, parrots, and hummingbirds), all possessing dedicated circuits for this behavior with marked similarities in their neuroanatomical organization [6, 21, 24]. Given these parallels, one might expect convergent similarities in the molecular organization of these circuits. Recent evidence supports a much closer relationship between parrots and passerines than previously recognized [34, 72], leading to the intriguing possibility that some molecular specializations of their vocal learning circuits may have evolved in a common ancestor. Indeed, recent studies have identified a number of shared molecular specializations in analogous vocal control nuclei across avian vocal learners [59]. Our demonstration that songbird novel genes have been incorporated into their unique vocal control nuclei suggests that in addition, these lineages also possess unique molecular specializations related to their particular vocal learning circuits. These specializations could relate to neuronal populations and connections unique to songbirds. For example, the songbird direct pre-vocal motor cortical projection to the basal ganglia, HVC-to-Area X, is absent in parrots [73], and possibly also in hummingbirds (Mello et al., unpublished). Alternatively, the roles played by songbird-unique genes could be subserved by functionally analogous genes in parrots and hummingbirds. Further study of avian vocal learners may reveal further group-specific specializations, as well as shared molecular features that may represent fundamental requirements for vocal learning.

## Conclusions

Our efforts resulted in: 1) A well-curated list of novel Zebra finch gene models, allowing for the identification and analysis of songbird-unique genes as well as improving the annotation of the Zebra finch genome by establishing orthology for hundreds of genes previously reported in error as being novel; 2) a refined map of synteny disruption sites likely representing chromosomal breakpoints in songbirds in comparison with non-songbird avian species and other vertebrates; 3) a set of songbird-unique genes that are transcriptionally active with expression patterns that diverge from those of parent genes, likely associated with unique aspects of songbird biology; and 4) a subset of songbird-unique genes that are specialized in nuclei of the vocal control system. These findings provide novel information on molecular processes that operate at the level of brain circuitry involved in vocal control, and represent prime candidate targets for gene manipulations of vocal learning.

## Methods

### Curation of ensembl models

We manually curated three distinct categories of gene models predicted in the genome of the Zebra finch (taegut3.2.4) by Ensembl’s Genebuild pipeline (e59; [47]). The first set consisted of the set of Ensembl models annotated as Uncharacterized Proteins with a status of ‘Novel’ in Zebra finch. We identified this set by retrieving the complete set of protein-coding models in Zebra finch from Ensembl BioMart (ensembl.org/biomart/martview), and excluding all models annotated as “Known”, models with orthologs in other species, and models placed onto ‘chromosome Unknown’, which are thought to largely represent allelic variants. The second set consisted of all genes identified by Ensembl as constituting Zebra finch-specific duplications based on the presence of a hyphen and a number following the gene symbol (e.g. *CASC1-1*, *CASC1-2*). We note that many of these genes’ status have changed to ‘novel’ or ‘uncharacterized’ protein in the latest release (e75) The third set included in our analysis consisted of models previously identified as belonging to expanded gene families in the initial description of the Zebra finch genome (see Supplementary Figure Three in [27]). Of note, we did not re-analyse the expanded gene sets for *PAK3* and *PIM1* that had been previously characterized [31].

To curate all three sets of genes and thus obtain a set of putative novel, duplicated, or expanded loci, we used the following steps: 1) We retrieved the complete nucleotide and protein sequences for each predicted model from Ensembl Biomart. 2) We BLAT-aligned [74] each model sequence to the genome assemblies of Zebra finch (taeGut1); two galliform genomes, chicken (galGal4) and turkey (melGal1); and five non-avian genomes of each major vertebrate lineage, namely a lizard (anoCar2), frog (xenTro3), Zebrafish (danRer7), mouse (mm10), and human (hg19) utilizing the UCSC genomic browser (genome.ucsc.edu, [75]). 3) We manually examined all high scoring (>50) hits within their genomic context, taking into account sequence quality and genome assembly gaps as well as comparing alignments to any existing annotations at these loci (e.g. human proteins mapped by chained tBLASTn, refSeqs from other species, and expressed sequence tags (ESTs)). This allowed us to identify some models as being artifactual due to misalignment or redundant alignment to known loci, excluding these from further analysis. In some cases, we also identified previously unrecognized paralogs for which no Ensembl model is currently available. These were added to our candidate novel gene set for further analysis. 4) To confirm the identity of each non-artifactual BLAT alignment of the models, we conducted a syntenic analysis in the UCSC browser, comparing the genes flanking each hit (at least three genes upstream and downstream) in Zebra finch to all other species of interest. This allowed us to exclude models whose syntenic placement revealed them to be known genes, including hits to known paralogs and related gene family members. We also excluded models for which we identified orthologs in species aside from Zebra finch, making note where possible of the “parent” gene orthologous between chicken and Zebra finch which may have been duplicated in songbirds to give rise to the novel genes examined further in this study (for example, see Figures 2, 3A). This approach allowed us to correctly annotate models based on a combination of sequence identity and synteny. It is also highly sensitive in discriminating paralogs from related gene family members, and in detecting additional loci not currently predicted by an Ensembl model. We have contacted Ensembl for incorporation of these corrected gene annotations into a future Zebra finch genome annotation release.

### Identification of songbird-unique genes

We next used a BLAST resource developed by BGI (phybirds.genomics.org.cn) to search for evidence of the models representing candidate novel songbird genes resulting from our curation effort (see preceding paragraph) in 45 new avian genomes (described in [34, 35], the Budgerigar genome used is further described in [76]). This includes basal ratites, galloanseriformes, and a range of shorebirds and landbirds, as well as other vocal learning groups (e.g. parrots, hummingbirds) and their sister taxa (falcons, swifts). We determined the number of hits of each model to each of these species, and identified models where passerines possessed additional gene copies not present in other avian species. This resource provides only the BLAST hits themselves with no genomic context, which makes it impossible to separate true hits from alleles, as well as to establish orthology among hits in different species. In order to address these limitations and to precisely identify the set of novel genes which arose in the songbird lineage, we examined the alignment and synteny of these Zebra finch models in genomes of critical comparative relevance to our goals, namely: two songbirds, Medium ground finch and American crow; two non-songbird passerines, Golden-collared manakin, a suboscine, and Rifleman, a New Zealand wren; and the nearest non-passerine relative, Budgerigar. To accomplish this task, we forged provisional annotations of these genomes by BLAT-aligning the complete Zebra finch Ensembl model set using a standalone server-based BLAT implementation with parameters replicating the web-based UCSC browser. We then BLAT-aligned our putative novel gene set with more sensitive parameters (e.g. allowing for more mismatches, returning lower-scoring alignments) in order to ensure that any trace of these models would be detected. We also BLAT-aligned the set of orthologous parent models from Zebra finch and chicken (in which predicted models are often more complete), in order to detect novel genes where the Zebra finch copy may not cross-align well to the other species due to genetic divergence. Finally, we imported these genomes and all described BLAT alignments into the Integrated Genomics Browser (IGV, [77]), a server-side alternative to the web-based UCSC browser. This procedure allowed us to examine the alignments of the candidate songbird-specific novel genes within the syntenic context of these genomes, enabling us to distinguish orthologs of known genes from novel genes. By identifying which novel loci were present in each species, we were also able to pinpoint their phylogenetic origin. We confirmed the accuracy of this method for establishing phylogenetic placement by replicating the results in the web-based UCSC browser for two species, Medium ground finch (geoFor1) and Budgerigar (melUnd1), which became available on the UCSC site midway through the study. No discrepancies were found between the two methods.

### Detection of Zebra finch chromosomal rearrangements by alignment of homologous synteny blocks in chicken and Zebra finch

To identify novel genes and/or duplications that might be associated with chromosomal rearrangements in Zebra finch, we used a previously established genomics approach [36] to first identify any breaks in gene synteny by analysing the order of appearance of orthologous gene pairs in the genomes of chicken and Zebra finch. To accomplish this, we used Ensembl BioMart [78] to retrieve the complete set of 11,132 genes from Ensembl that have been predicted to be of type “ortholog_one-to-one” in both Zebra finch (taeGut3.2.4) and Chicken (WASHUC2, May 2006). We only included genes with known physical locations in both genomic assemblies. We then used SyntenyTracker [79] in Orthologous Gene Pair mode with default settings (i.e. distance between markers 1 Mb, block size 0 bp, block length 2 Mb, jumping distance 2 Mb, reference genome “Zebra finch,” target genome “chicken”) to identify Homologous Synteny Blocks (HSBs) for each orthologous chromosome pair. Here we define HSBs as a continuous block of two or more adjacent homologous genes that appear without interruption, and on the same chromosomes in the two species being compared. To verify the results of SyntenyTracker, and further refine our breakpoint analysis, we also aligned the entire set of orthologous gene pairs according to their relative position in the Zebra finch genomic assembly, and then scanned the alignments to search for cases where two or more genes appeared with the correct gene order, but in different positions on the chromosome (e.g. translocation), or where the gene order was reversed (e.g. inversion). We retrieved from Biomart a subset of Zebra finch genes, which included known and novel protein coding genes, as well as non-coding and pseudogenes (e.g. miRNAs, snoRNAs) that have no known orthologs in chicken. Paired orthologous and non-orthologous genes were then sorted according to their relative chromosomal positions within the Zebra finch assembly to identify genes lying within a predicted chromosomal “breakpoint”, or gap in genomic sequence between two consecutive HSBs. To determine which of the syntenic rearrangements between Zebra finch and chicken had occurred in the songbird or the chicken lineage, we also compared the syntenic regions flanking the SD sites to mammalian species that have well-assembled and curated genomes (i.e., mouse and humans) as well as to lizard when the corresponding regions were well-assembled.

### Protein coding domain motif analysis

To determine whether novel and duplicated genes contain or might be missing specific protein coding domains that specify their possible molecular function, we analysed each novel gene’s protein coding sequences with Interproscan5 [80] using default search parameters and all available protein domain definitions (e.g. SUPERFAMILY, SMART, PfamA, PROSITE). For genes that had no Ensembl model, we analysed the most complete open reading frame prediction available in the songbird genomes. We paid particularly close attention to the duplicated genes, since any differences between these and their parent genes would suggest a possible divergence in protein function. Schematic representations of predicted protein domain structures were designed using Prosite MyDomains (prosite.expasy.org/mydomains/).

### Analysis of songbird novel gene expression

To explore the expression of genes of interest, we searched for Zebra finch brain-derived cDNA clones from ESTIMA [81], SongbirdTranscriptome.net [82], or the Rockefeller database [57], as well as clones derived from several additional songbird tissues [37], that were aligned to novel gene loci in the UCSC genome browser. For cases where the novel gene represented a songbird duplication or expansion we searched for cDNAs for both the expanded copies and suspected ortholog. To maximize the likelihood that the resulting probe would be specific to a given locus, we selected clones containing primarily 3’-untranslated sequence, minimizing the inclusion of protein coding regions that might be conserved among close paralogs and/or related gene family members. In the case of TMRA, we also fully sequenced the cDNA clone to define its 3’-end and establish the presence of a polyadenylation tag (polyA). In the case of YTHDC2L1, we identified a single clone (CK309358) that contains a 3’-UTR exon (Exon C5; Additional file 2: Figure S2B) that uniquely maps to the locus containing YTHDC2L1. This unique exon, which contains the polyA tag, constitutes ~30% of the total length of the probe used for hybridizations. To more directly establish clone specificity, we BLAT-aligned the complete nucleotide sequence for each clone to the Zebra finch genome, and analysed the resulting hits. In cases where a clone aligned to multiple loci, we attempted to identify the locus that generated that clone by determining the BLAT query that produced the largest alignment (e.g. number of exons) with the highest percent identity to the aligned region, as well as identifying any locus-specific segments in the clones. Additional confirmation of songbird novel gene expression was obtained by analysing previously published Zebra finch RNA-seq data from several tissues; spleen, muscle, skin, liver, testes, and whole embryo [27]; available through NCBI with BioProject accession code PRJNA17289. Only reads that mapped to unique locations, and which overlapped with novel gene exons were considered.

### Brain preparation for *in situ*hybridization

The preparation of tissue for the analysis of brain expression is as described in [33]. This study used 10 adult male Zebra finches that were bred and housed at OHSU in accordance with IACUC guidelines. Birds were moved into sound-attenuated chambers the evening before sacrifice and monitored for 1 hour following lights-on to ensure that they were non-singing to reduce variation due to hearing- or singing-induced gene regulation in song nuclei and adjacent regions [83, 84]. Following decapitation, brains were removed and flash frozen in Tissue-tek embedding medium on a dry ice-isopropanol slurry in under 5 minutes to ensure sufficient RNA quality. Brains were cut into 10 μm sections on a Leica CM1850 cryostat, placed onto glass slides, fixed for 5 minutes in a 3% paraformaldehyde fixative solution, and stored at -80°C until further use.

### Analysis of brain expression by *in situ*hybridization

We followed a previously established protocol for optimized detection of gene expression through non-radioactive *in situ* hybridization [33]. Briefly, for each brain-derived cDNA clone, we generated digoxygenin-labelled sense and antisense riboprobes, performed in situ hybridization using high-stringency hybridization and wash conditions, and detected cellular labelling by immunohistochemical detection with alkaline phosphatase precipitation. Under these hybridization conditions, sense-strand hybridizations and hybridizations that are performed without probe give no signal. Replicates were run for each probe on at least two adjacent sections (n = 3 brains) for all of the major song nuclei. Resulting high-quality sections were then imaged for digital analysis at 0.42 μm/pixel with an Olympus Nanozoomer. For each nucleus we assessed relative level of brain expression based on visual assessment and a scoring scale from low to high. Genes that we found to be expressed in song nuclei were qualitatively analysed for enrichment (or impoverishment) relative to surrounds (e.g. RA vs. arcopallial shelf) by at least two independent observers.

## Electronic supplementary material

Additional file 1: **Gene names which appear in parentheses represent current gene annotations which have been updated by Ensembl since our initial analyses took place.** **Table S1.** putative gene duplications and expansions which correspond to partial models of known genes. In the process of the BLAT annotation step of the syntenic analysis, some additional novel models were opportunistically detected as partial models and added to Table S1. **Tables S2-S3.** putative gene duplications and expansions corrected to known genes or found to have non-songbird orthologs. **Table S4.** putative novel genes corrected to known genes or found to have non-songbird orthologs. **Table S5.** gene expansions not shared by all songbirds, or shared by non-songbird outgroups, as revealed by phylogenetic analysis; †: novel gene, no obvious parent gene, *: cannot determine which locus constitutes the parent gene. **Table S6.** refined set of syntenic disruptions (SDs) in Zebra finch relative to chicken. Red text indicates that the specific position of genes within the SD (whether they were at the start, end, within, or flanking the SD) were refined during manual curation. Thick black lines indicate tandem SDs. Green highlights SDs specific to the Zebra finch lineage. Pink highlights SDs specific to chicken lineage. Grey highlights SDs which could not be definitively assigned to either lineage. (XLSX 148 KB)

Additional file 2: Figure S1: Schematic alignment of brain-derived cDNAs with specific *CASC1* duplicate loci. **Figure S2.** percent identity (A) and schematic alignment (B) of brain-derived cDNAs with specific *YTHDC2* expanded loci. **Figure S3.** (A) example of an Ensembl gene prediction which splits a gene across two partial models, (B) example of a misannotated Ensembl gene prediction. (PDF 491 KB)
