# Supplementary material for: Comparative genomics reveals molecular features unique to the songbird lineage
Source: BMC Genomics. 2014 Dec 13;15(1):1082. doi: 10.1186/1471-2164-15-1082 (PMC4377847; doi:10.1186/1471-2164-15-1082)

Figure S1: schematic alignment of brain-derived cDNAs with specific CASC1 duplicate loci

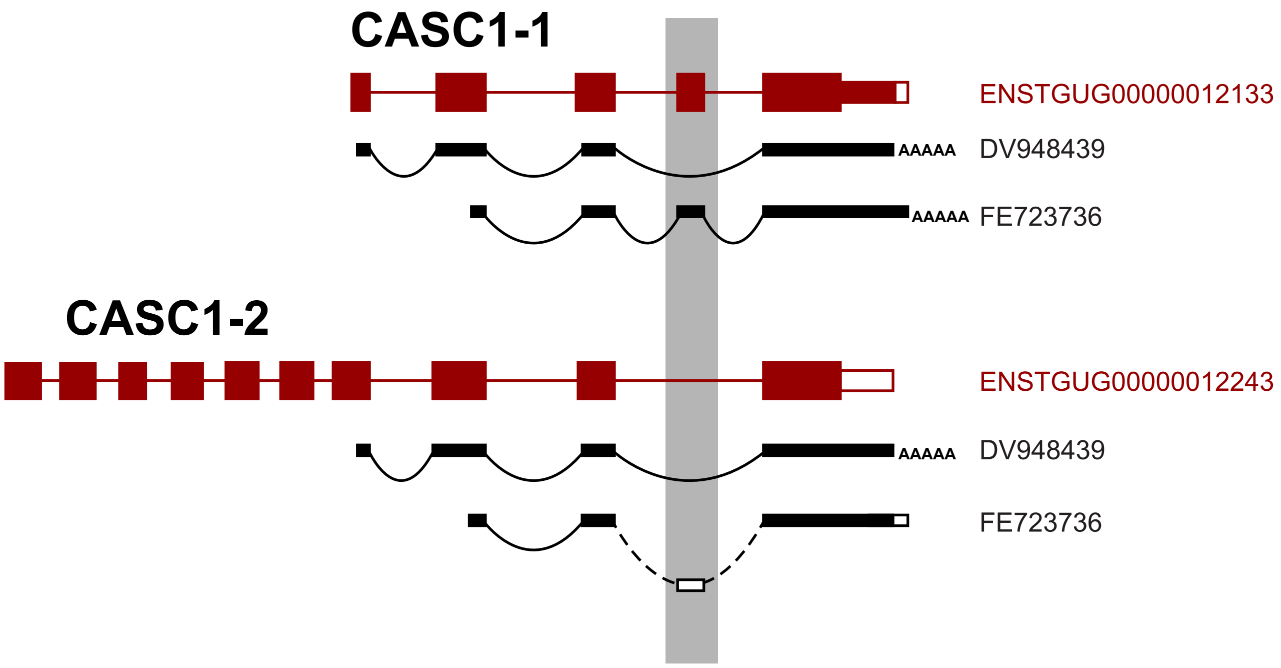

Figure S2: percent identity and schematic alignment of brain-derived cDNAs with specific YTHDC2 expanded loci

A

DV946054

Gene	Segment/Exon D1	D2	D3	D4	D5	D6	D7
YTHDC2							
YTHDC2L1		84.1					
YTHDC2L2		85.2	87.1				
YTHDC2L3		91.8	93.2	95.8	93	93.5	91.2
YTHDC2L4			82		92.7	89.7	85.7
YTHDC2L5	100	100	100	100	100	100	100
YTHDC2L6			86.7				

CK309358

Gene	Segment/Exon C1	C2	C3	C4	C5
YTHDC2	96.3				
YTHDC2L1	100	100	99.5	100	100
YTHDC2L2	100	99.1	97.5	99.1	
YTHDC2L3		94.9			
YTHDC2L4	100	90.6			
YTHDC2L5		92.2			
YTHDC2L6				97.3	

B

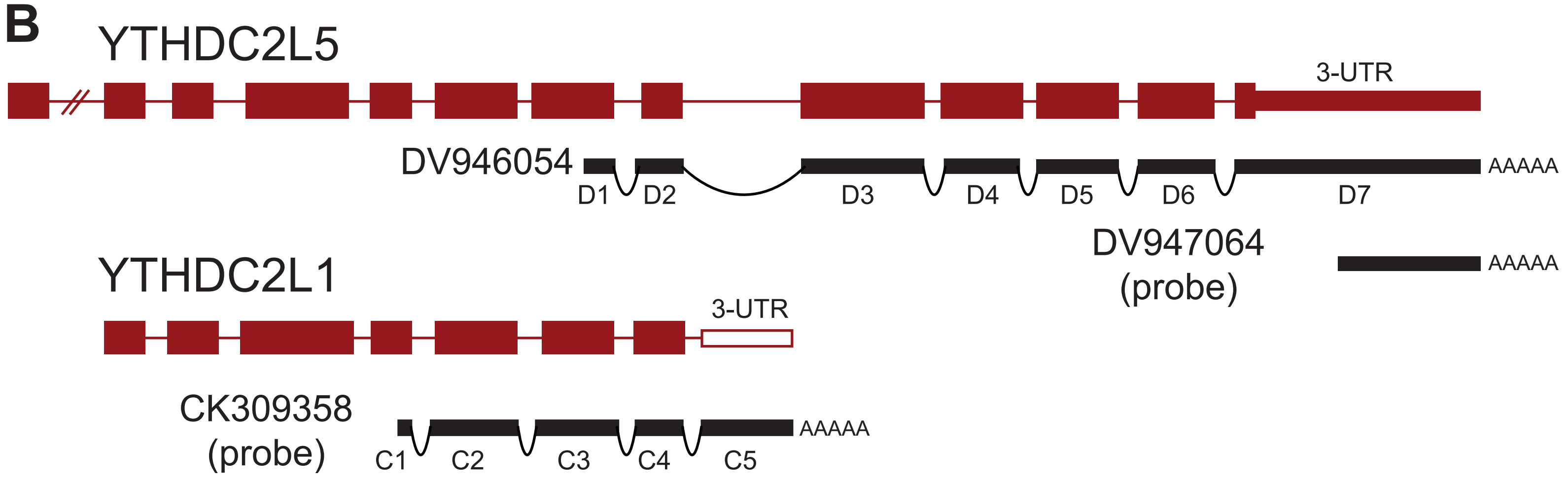

Figure S3A: example of an Ensembl gene prediction which splits a gene across two partial models

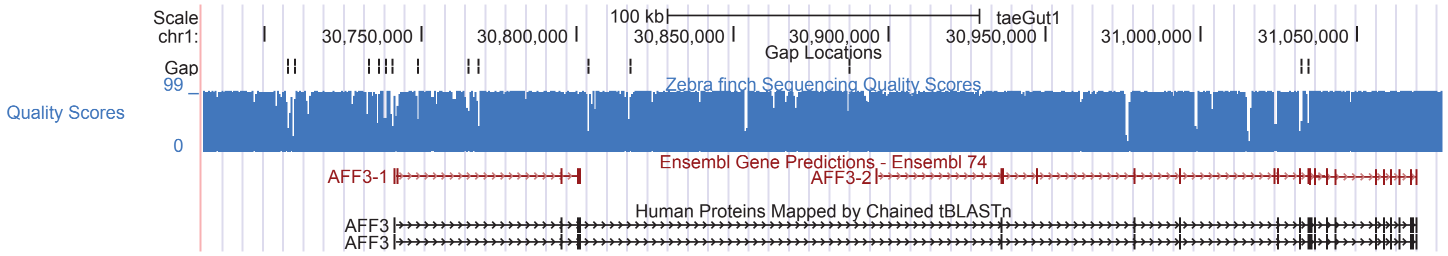

Figure S3B: example of a misannotated Ensembl gene prediction

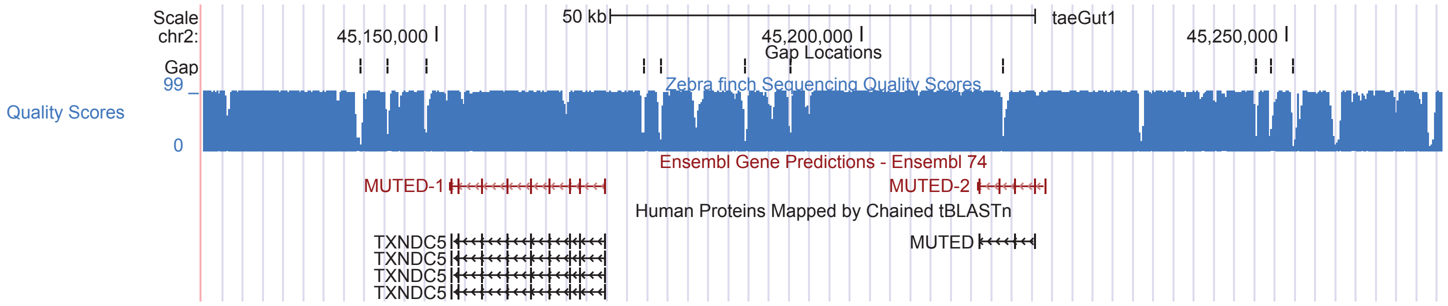

Supplement: Supplementary file 2 — Additional file 2: Figure S1: Schematic alignment of brain-derived cDNAs with specific CASC1 duplicate loci. Figure S2. percent identity (A) and schematic alignment (B) of brain-derived cDNAs with specific YTHDC2 expanded loci. Figure S3. (A) example of an Ensembl gene prediction which splits a gene across two partial models, (B) example of a misannotated Ensembl gene prediction. (PDF 491 KB) [file 12864_2014_6805_MOESM2_ESM.pdf]
